# Could parental awareness of DBR influence youth off-campus sports? Test of a conditional process model

**DOI:** 10.1186/s12889-023-16873-4

**Published:** 2023-10-21

**Authors:** Shuying Tan, Hu Lou, Xue Liu, Jin Chen

**Affiliations:** School of Sports Science, Nantong University, Nantong, 226019 Jiangsu Province China

**Keywords:** Double reduction, Parents, Education anxiety, Exercise attitude, Sports

## Abstract

**Background:**

The “Double Reduction” policy published in China provides a new opportunity to increase youth sports. Based on the perspective of parents’ influence on their children, this study aimed to explore the impact of parental awareness of the “Double Reduction” policy on youth off-campus sports.

**Methods:**

This study was conducted empirically through a nationwide sample survey to analyze the relationship between parental awareness of the “Double Reduction” policy, parental education anxiety, and parental attitudes toward children’s participation in sports and youth off-campus sports to construct and verify a conditional process model.

**Results:**

Parental awareness of the “Double Reduction” policy had a significant positive impact on their children’s off-campus sports (*β* = *0.103, SE* = *0.018, P* < *0.01*), but had a significant negative impact on parental education anxiety (*β* = *-0.305, SE* = *0.032, P* < *0.01*) which showed a significant negative effect on their children’s off-campus sports (*β* = *-0.114, SE* = *0.011, P* < *0.01*) and played a significant intermediary role between parental awareness of the “Double Reduction” policy and children’s off-campus sports (*β* = *0.031, 95%CI: 0.020* ~ *0.044*). All dimensions of parental education anxiety showed different characteristics with only academic achievement anxiety (*β* = *0.034, 95%CI: 0.021* ~ *0.047*), parent–child interaction anxiety (*β* = *0.027, 95%CI: 0.014* ~ *0.038*), and learning attitude anxiety (*β* = *0.024, 95%CI: 0.015* ~ *0.033*) presenting significant mediating effects. The interaction between parental education anxiety and parental attitudes toward children’s sports significantly positively impacted youth off-campus sports (*β* = *0.008, SE* = *0.001, P* < *0.01*)*.* The moderating role of parental attitudes toward their children’s participation in off-campus sports, indicating it was significant (*95%CI: 0.005* ~ *0.011*) along the pathway of parental awareness of the “Double Reduction” policy to parental education anxiety to youth off-campus sports.

**Conclusions:**

Parental awareness of the “Double Reduction” policy has a positive impact on youth off-campus sports. Parents’ anxiety about their children’s academic achievement, parent–child interaction anxiety, and learning attitude anxiety mediate in the process model. Parental attitudes toward sports can magnify the relationship between their education anxiety and youth off-campus sports.

## Introduction

Maintaining adequate physical activity is an important way for teenagers to prevent and control myopia, shape personalities, and promote healthy development [1]. However, a recent survey which was conducted by the World Health Organization reported that more than 80% of adolescents are not playing enough sports [2]. The promotion of youth sports is one of the current focal points of social concern and academic research. In China, the General Office of the CPC Central Committee and the General Office of the State Council issued the “Double Reduction” policy (referred to as DBR) in 2021 that aims to reduce the homework burden and off-campus training burden of students in compulsory education. The policy clearly states that the quality of after-school services should be improved, sports, art, labor, club activities and so on should be carried out, and a good educational environment should be built to promote the all-round development and healthy growth of students [3]. Therefore, DBR provides new opportunities for the development of off-campus sports and is expected to provide more adequate time and energy for youth off-campus sports. There is a positive consensus among scholars in the field of sports that DBR promotes both on-campus and off-campus sports for young people [3]. However there has been little research on DBR since its implementation one year ago, and its impact mechanism is still unclear. Parents are very close people to children and their cognition, attitude, and behavior will have an impact on many aspects of their children [4]. For instance, when parental education anxiety is high, it may push the child to spend more time studying and attending after-school classes to achieve the ideal score, resulting in affecting the child’s sports to a certain extent. In addition, when parents do not support their children to play sports, it may also affect their children’s off-campus sports. Thus, based on the perspective of parents’ influence on their children, this study aimed to explore the impact of parental awareness of DBR on youth off-campus sports, as well as the role of parental education anxiety and parental attitudes toward children’s sports in the influence process through a nationwide sample survey, to provide a reference for the further promotion of teen sports after the implementation of DBR.

### Parental awareness of DBR and youth off-campus sports

Off-campus sports refer to the sports that students take part in after school and outside the school, which are vital forms of activity to promote the healthy growth of teenagers [5]. The purpose of DBR is to reduce the burden of assignments and prohibit strictly supplementary classes in subjects to provide time for teenagers to do sports outside of school. DBR reshapes the education ecology of healthy growth and provides development space for youth sports as well [6]. Therefore, after DBR was conducted, some studies [7, 8] believe that DBR has been seen as a new “opportunity” to promote youth participation in sports from different research perspectives. As DBR is directed at the compulsory education stage, where students are relatively young, parents’ perceptions are crucial to the effectiveness of the policy [9]. Thus, this study used parental awareness of the policy as the independent variable of the hypothesis model. Sport has already been expected to be an important content to show the effect of DBR due to its many benefits to youth development [10]. From the perspective of performance evaluation of public policies, the degree to which policy is stimulating individuals can be measured by their perception of the intensity of the policy [11]. As mentioned above, DBR will provide a new opportunity to improve off-campus sports for teenagers. This study proposes hypothesis H1: Parental awareness of DBR has a significant positive effect on their children’s participation in off-campus sports.

### Parental education anxiety and parental attitudes toward their children’s sport

Parental education anxiety refers to their worries about their children’s education, including anxiety about their children’s learning attitude, school choice, future development, academic achievement, and parent–child learning interaction [12]. DBR aims not only to ease the burden on students and promote their healthy development but also to reduce the burden and pressure on parents. According to a survey conducted by the Central Committee of the Communist Youth League, the education anxiety of parents of students in compulsory education has been alleviated after the implementation of DBR [13]. Based on this, hypothesis H2 is proposed: Parental awareness of DBR has a significant negative influence on parental education anxiety. Nowadays, parental education anxiety is a widespread negative emotion among parents in China and has a profound influence on adolescents’ learning, life, and health [14]. Relevant studies have also shown that parental education anxiety is detrimental to their children’s off-campus sports [4]. Thus, we further propose hypothesis H3: Parental education anxiety has a significant negative influence on their children’s off-campus sports. In addition, parents’ awareness of the event intensity of DBR affects their children’s after-school sports, which may be achieved through the intermediary of parental educational anxiety. So we predict that parental awareness of the event intensity of DBR possibly reduces parental educational anxiety. Subsequently, the reduction of parental educational anxiety may increase the off-campus sports among young people. Thus, we continue to propose hypothesis H4: Parental education anxiety plays a mediating role between parental awareness of DBR and their children’s off-campus sports.

**Fig. 1:**
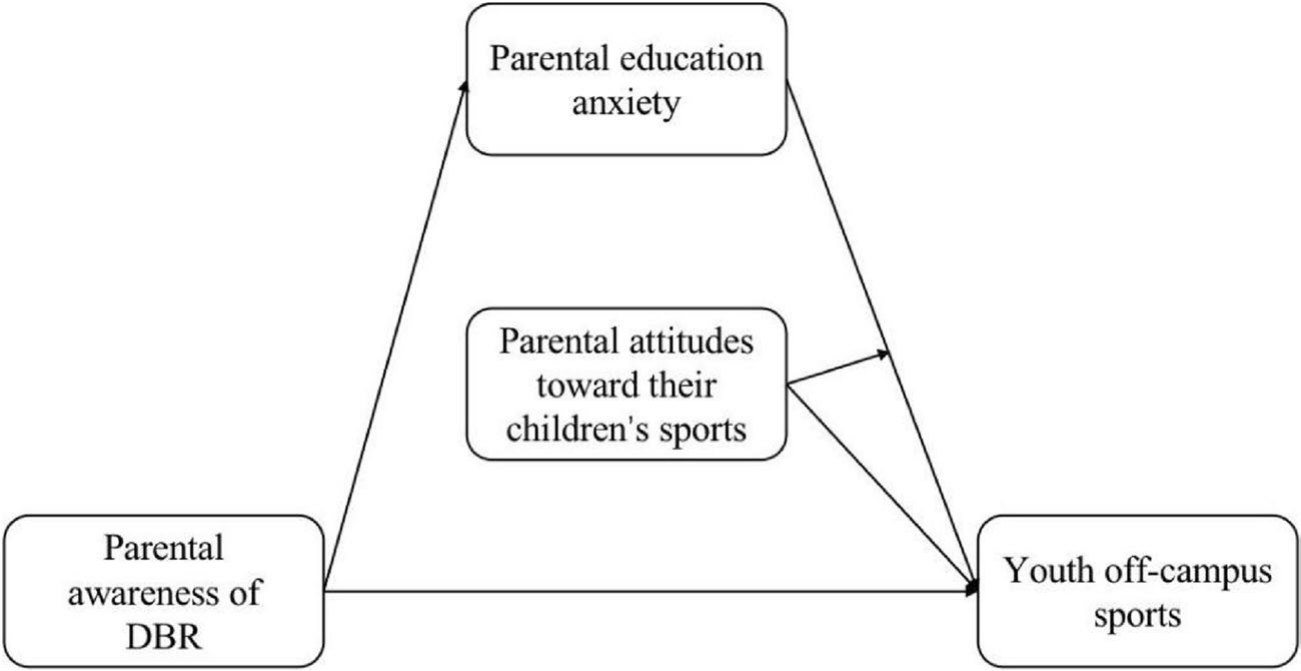
Hypothetical model of parental awareness of DBR impact on youth off-campus sports

There is a positive correlation among parental attitudes toward their children’s participation in sports, youth participation in sports, and sports behaviors [15, 16]. Parents’ positive attitudes toward their children’s participation in sports have the ability to amplify the impact of reduced parental educational anxiety on youth extra-school sports. In contrast, if the level of parents’ attitudes is low, the relief of parental educational anxiety may not bring teenagers a higher level of off-campus sports. Consequently, we hypothesize that: H5: Parental attitudes toward their children’s participation in sports can significantly and positively affect youth off-campus sports. H6: Parental attitudes toward their children’s participation in sports play a moderating role between parental education anxiety and youth off-campus sports. The hypotheses for the relationship among parental awareness of DBR, education anxiety, attitudes toward exercise, and youth off-campus sports are shown in Fig. 1.

Based on the above schematic, this study explores the effects of parental awareness of DBR on youth off-campus sports, the mediating role of parental education anxiety in the impact of parental awareness intensity of DBR on youth sports, and the moderating role of parental attitudes toward their children’s participation in sports in the relationship between parental education anxiety and youth sports.

## Research methods

### Research objects

We adopted the method of stratified random cluster sampling to comprehensively consider the three major economic belts in the east, middle, and west of China. And we selected three cities in each economic zone according to high, medium, and low GDP (in the east: Nantong, Jiangsu Province, Lishui, Zhejiang Province, and Fuxin, Liaoning Province; in the middle: Taiyuan, Shanxi Province, Puyang, Henan Province, and Xiangxi Tujia and Miao Autonomous Prefecture, Hunan Province; in the west: Chongqing, Yibin, Sichuan Province, and Yili, Xinjiang Uygur Autonomous Region). From March to April 2022, 100 paper questionnaires were distributed on site in each grade of 9 junior middle schools, 2,700 of which were distributed and 2,679 were recovered, including 2,420 valid questionnaires (recovery rate = 89.6%). The valid data included 1,190 male students and 1,230 female students with a basically balanced gender ratio. There were 826 students in Grade 7, 913 students in Grade 8 and 681 students in Grade 9 (grade distribution: χ^2^ = 3.74, *P* = 0.15).

### Measuring tools

#### Parental awareness of the intensity of DBR

We administered the Chinese version of the Event Intensity Awareness Scale revised in combination with DBR [17]. There are 11 items in total, including 4 reverse scoring items, using the Likert 7-point scoring method, in which 1 represents “completely disagree” and 7 represents “completely agree.” And higher scores indicate higher parental awareness of DBR. The 11 items include three dimensions of novelty, criticality, and disruption awareness of DBR. The dimensions show high reliability (Cronbach’s α = 0.870, 0.846, and 0.858, respectively). Confirmatory factor analyses (*X*^*2*^* / df* = 4.924 < 5, *RMSEA* = 0.041 < 0.08, *NFI* = 0.982 > 0.9, *IFI* = 0.985 > 0.9, *TLI* = 0.980 > 0.9, *CFI* = 0.985 > 0.9) further confirm high structural validity.

#### Parental education anxiety

We used the Parents’ Education Anxiety Questionnaire [12] containing 21 items in total on a Likert 5-point scale, in which 1 represents “completely disagree” and 5 represents “completely agree.” Higher scores indicate higher parental anxiety about their children’s education. Three items of the questionnaire reflect children’s learning attitudes anxiety, three items reflect school choice anxiety, four items reflect future development anxiety, four items reflect academic achievement anxiety, and four items reflect parent–child learning interaction anxiety. The five dimensions show high reliability (Cronbach’s α = 0.785, 0.782, 0.809, 0.811 and 0.849, respectively). Confirmatory factor analyses (*X*^*2*^* / df* = 4.410 < 5, *RMSEA* = 0.038 < 0.08, *NFI* = 0.974 > 0.9, *IFI* = 0.980 > 0.9, *TLI* = 0.975 > 0.9, *CFI* = 0.980 > 0.9) indicate good structural validity.

#### Parental attitudes toward their children’s participation in sports

We used the Parental Sports Attitude questionnaire [18] which contains 9 total items on a Likert 5-point scale, in which 1 represents “completely disagree” and 5 represents “completely agree.” Higher scores indicate more positive parental attitudes toward their children’s physical exercise. The questionnaire shows high reliability (Cronbach’s α = 0.921) and good structural validity (*X*^*2*^* / df* = 4.097 < 5, *RMSEA* = 0.036 < 0.08, *NFI* = 0.992 > 0.9, *IFI* = 0.994 > 0.9, *TLI* = 0.992 > 0.9, *CFI* = 0.994 > 0.9).

#### Youth off-campus sports

We finally used the Physical Activity Rating Scale [19] revised with emphasis on “off-campus”. The scale includes 3 total items combining the frequency of weekly off-campus sports, the duration of each off-campus sport, and the intensity of ordinary off-campus sports. The total score was the product of the three. And the answers to each item are divided into 5 levels, with frequency and intensity scored on a scale of 1–5, duration scored on a scale of 0–4, and total scored on a scale of 0–100, with higher scores indicating higher levels of off-campus sports among young people. Reliability and validity analyses showed high scores for both (retest reliability* r* = 0.82, internal consistency reliability: Cronbach’s α = 0.907).

### Data analysis

SPSS25.0 was used for descriptive statistics and correlation analysis of variables, and linear regression analysis was used for the main effect test. Process 4.0 plug-in is used to analyze the mediation effect and regulatory effect. Model 4 was used to test the mediating effect of parents’ educational anxiety, and model 59 was used to test the moderating effect of parents’ attitudes. The bootstrap method based on 2,000 resamples was used to examine the significance of the direct and indirect effects.

## Results

### Common method bias

Firstly, we collected data by self-reporting. Secondly, we used Harman’s single-factor test to test the possibility of common method bias before data analysis, where all variables were analyzed using non-rotating principal component analysis. The results indicate that there are no serious common method bias problems in this study, where the first factor explains 22.86% of the total variance, which is less than the critical value of 40%.

### Descriptive statistics and correlation analysis

The descriptive statistics showed that parental awareness of DBR had a certain degree of intensity and educational anxiety. The majority of teenagers had participated in some form of off-campus sports, with the highest number of them having “2 to 3 times a month” was 934 (39%), 753 (31%) having “1 to 2 times a week”, 517 (21%) having “3 to 5 times a week” and 96 (4%) “about 1 time a day”. In terms of intensity, the highest proportion of young people played “medium intensity” sports (939, 39%), followed by “high intensity” (480, 20%). More than three-quarters of adolescents played sports for less than thirty minutes each time, with 527(22%) playing “less than 10 min”, 706 (29%) playing “11–20 min” and 768 (32%) playing “21–30 min”.

The correlation analysis presented that parents’ awareness of DBR was significantly negatively related to parental education anxiety and positively related to youth off-campus sports. And parental education anxiety was significantly negatively related to parental attitudes toward their children’s participation in sports and youth off-campus sports. On the contrary, parental attitudes toward their children’s participation in sports were significantly positively related to youth off-campus sports. The relationship among these variables supports the test of subsequent hypotheses. The results are shown in Table 1.

**Table 1:** Descriptive statistics and correlation analysis results of awareness variables

	Variables	M	SD	1	2	3	4	5	6
1	Gender	-	-	1					
2	Stage	-	-	-0.003	1				
3	DBR awareness	33.94	7.83	-0.010	-0.056^**^	1			
4	Education anxiety	2.49	0.62	-0.011	0.054^**^	-0.305^**^	1		
5	Parental attitude	2.886	0.960	0.021	-0.011	0.103^**^	-0.063^**^	1	
6	Sports	8.670	6.859	0.010	0.040	0.145^**^	-0.150^**^	0.092^**^	1

^*^P < 0.05

^**^P < 0.01

However, there was some variation in the scores of the dimensions of parental education anxiety (Table 2), with their anxiety about parent–child learning interaction (2.79 ± 0.930) being the lowest, followed by anxiety about their children’s academic achievement (2.80 ± 0.878), anxiety about adolescent’s learning attitude ranking the third place (2.86 ± 0.911), adolescent’s future development anxiety ranking the forth place (3.04 ± 0.834), and school choice anxiety was the highest (3.11 ± 0.750). Due to the obvious differences, each dimension was analyzed separately and all dimensions were significantly negatively correlated with the awareness of DBR, except for the anxiety about the choice of school of teenagers which was significantly positively related to the awareness of DBR (*r* = *0.061, P* < *0.01*). In addition, all anxiety dimensions were significantly negatively correlated with sports.

**Table 2:** Scores of all dimensions of parental educational anxiety

Variables	M	SD
Academic achievement anxiety	2.80	0.878
Parent–child interaction anxiety	2.79	0.930
Learning attitude anxiety	2.86	0.911
Future development anxiety	3.04	0.834
School choice anxiety	3.11	0.750

### Main effects test

We found that parental awareness of DBR had a significant impact on their children’s off-campus sports (*β* = *0.103, SE* = *0.018, P* < *0.01*), consistent with our prediction H1. We also found evidence in favor of the remaining hypotheses that parental awareness of DBR had a significant negative effect on parental education anxiety (*β* = *-0.305, SE* = *0.032, P* < *0.01*), consistent with prediction H2, and parental education anxiety showed a significant negative effect on their children’s off-campus sports (*β* = *-0.114, SE* = *0.011, P* < *0.01*), consistent with prediction H3. What’s more, parental attitudes toward their children’s participation in sports had no significant impact on youth off-campus sports (*β* = *0.059, SE* = *0.016, P* = *0.074*), in contrast to H5 (Table 3).

**Table 3:** Main effects hypothesis test

Variables	Parental anxiety			Sports		
	*β*	*SE*	*P*	*β*	*SE*	*P*
DBR awareness	-0.305^**^	0.032	0.000	0.103^**^	0.018	0.000
Parental anxiety				-0.114^**^	0.011	0.000
Parental attitude				0.059	0.016	0.074
*R*^*2*^	0.093					
*F*	248.499					

^*^P < 0.05

^**^P < 0.01

### Mediating and moderating effects testing

The mediating effects of the test results are shown in Table 4. When education anxiety was entered as the independent variable, the total effect was significant (*β* = *0.127, 95%CI: 0.092* ~ *0.162*), and both the direct (*β* = *0.096, 95%CI: 0.060* ~ *0.132*) and indirect effects (*β* = *0.031, 95%CI: 0.020* ~ *0.044*) were significant, suggesting that parental education anxiety played a partial mediation role between parental awareness of DBR and their children’s off-campus sports, supporting prediction H4. While, when calculated separately for each dimension of educational anxiety, only the indirect effects of academic achievement anxiety (*β* = *0.034, 95%CI: 0.021* ~ *0.047)*, parent–child interaction anxiety (*β* = *0.027, 95%CI: 0.014* ~ *0.038*) and learning attitude anxiety (*β* = *0.024, 95%CI: 0.015* ~ *0.033*) were consistent with the hypothesis.

**Table 4:** Bootstrap test results of mediation effects

Effect type	Effect value	SE	95%CI
Total effect	0.127	0.018	[0.092, 0.162]
Mediating variables: education anxiety			
Direct effects	0.096	0.018	[0.060, 0.132]
Indirect effects	0.031	0.006	[0.020, 0.044]
Mediating variables: academic achievement anxiety			
Direct effects	0.093	0.019	[0.057, 0.129]
Indirect effects	0.034	0.006	[0.021, 0.047]
Mediating variables: parent–child interaction anxiety			
Direct effects	0.100	0.018	[0.064, 0.137]
Indirect effects	0.027	0.006	[0.014, 0.038]
Mediating variables: learning attitude anxiety			
Direct effects	0.103	0.017	[0.070, 0.137]
Indirect effects	0.024	0.005	[0.015, 0.033]
Mediating variables: future development anxiety			
Direct effects	0.124	0.018	[0.088, 0.160]
Indirect effects	0.003	0.006	[-0.009, 0.015]
Mediating variables: school choice anxiety			
Direct effects	0.149	0.018	[0.114, 0.184]
Indirect effects	-0.022	0.005	[-0.033, -0.012]

The interaction between parental educational anxiety and attitudes toward their children’s participation in sports showed a significant effect on youth off-campus sports (*P* < *0.01*), supporting prediction H6. We distinguished between “high” and “low” groupings by designating as one standard deviation above (M + SD) and below the mean (M-SD), respectively, to perform a simple slope test. The results showed that the effect of parental educational anxiety on youth off-campus sports was stronger under the condition of high parental attitudes (*k* = *1.791*) and weaker under the condition of low parental attitudes (*k* = *1.470*).

We further tested the moderating role of parental attitudes toward their children’s participation in off-campus sports, indicating that it was significant (*95%CI: 0.005* ~ *0.011*) along the pathway of parental awareness of DBR → parental education anxiety → youth off-campus sports. However, the attitude had a significant positive moderating effect on academic achievement anxiety (*95%CI: 0.015* ~ *0.035*) and parent–child interaction anxiety (*95%CI: 0.014* ~ *0.032*) among the effective mediators of the dimensions of education anxiety (Table 5).

**Table 5:** Moderating effects of parental attitudes toward their children’s exercise

Paths	*β*	*SE*	*95%CI*
DBR awareness → parental education anxiety → youth off-campus sports	0.008	0.001	[0.005, 0.011]
DBR awareness → academic achievement anxiety → youth off-campus sports	0.025	0.005	[0.015, 0.035]
DBR awareness → parent–child interaction anxiety → youth off-campus sports	0.023	0.005	[0.014. 0.032]
DBR awareness → learning attitude anxiety → youth off-campus sports	-0.008	0.005	[-0.018, 0.001]

## Discussion

### Parental awareness of DBR and its effects

We found that parental awareness of DBR had a significant negative effect on parental educational anxiety and a significant positive effect on their children’s off-campus sports by analyzing data from the nationwide sample. The reason for this may be that the introduction of DBR reflects the party’s and the nation’s macro-adjustment to reduce the burden of homework on teenagers and the firm determination to govern off-campus training [20]. If parents are aware of the importance of DBR in the current educational system, and the purpose and a series of measures of the education authorities to reduce the burden on students, they may relieve the stress and anxiety about their children’s education. And when parents realize the importance of DBR, it will promote the necessity of youth sports, let parents cooperate with the school to ease the burden of assignment and extra-curricular training for young people and reshape the system of off-campus sports [21]. In terms of parent–child influence theory, the interpersonal relationship established by the interaction between parents and children in the family is the earliest social relationship to which people are exposed and which affects individual personality development, social awareness, behavior, and many other aspects [22]. Because of the young age of compulsory school students, the family is the most important micro-environmental system to which they are exposed, and the important members of the system can have a profound influence on the behavior of teenagers [8]. Thus, parental awareness of DBR can influence their children’s off-campus sports. Encouraging and promoting youth physical activity through policy adjustments is very effective overseas, and the investigation of DBR in this study supports the theoretical view as well. Although there is little empirical research to support the idea that youth sports will increase after DBR, some studies [8] theorize that DBR may bring opportunities to increase students’ off-campus physical activity, which is consistent with the findings we obtained here.

### The mediating role and differences of education anxiety

Our results showed that parental education anxiety negatively affected youth off-campus sports, consistent with the prior research [23] which also argues that the marginalization of physical education is caused by the utilitarian educational mentality of Chinese parents and the educational anxiety it brings. Only by reducing this anxiety can they untie their children and let their children participate in sports which are beneficial to children’s healthy growth and comprehensive and free development [24].

The levels of parental educational anxiety in different dimensions vary in scale with respect to each other. For example, parental anxiety about parent–child interactions is seemingly less significant than anxiety about adolescents’ academic achievement, which is in turn less important than anxiety about adolescents’ learning attitudes, future development, and adolescents’ choice of school for higher education. We analyzed each dimension of education anxiety separately as a mediating variable to further explain the mechanism and features of the effect of DBR on youth off-campus sports. The results revealed that anxiety about academic achievement, parent–child interaction, and anxiety about learning attitude mediated the effect of parental awareness of DBR on youth off-campus sports.

At present, children’s poor performance can trigger varying degrees of anxiety for many parents in China. If children do not spend all their time studying, parents will feel insecure. And once the children’s performance drops, the parents will become helpless and furious [25]. Many children receive tiger parenting at an early age in order to get good grades. Education becomes a strange pattern of constant repetition of homework exercise, a busy schedule of remedial classes but fewer extracurricular activities such as sports. This notion of academic achievement as the core goal is a departure from true education. DBR alleviates parental anxiety about their children’s academic achievement by reducing the burden of homework and extracurricular training on students, which in turn improves youth sports.

Due to the distorted perspective of education, anxiety over the education of their children is overriding the normal life of the family. Meanwhile, parents put too much pressure on their children, when they are overly committed to education, which can cause bad feelings among teenagers and even affect the parent–child relationship [15]. DBR reduces the burden on students and stress on parents, and can relieve the anxiety about the relationship between parents and children. Parent–child relationships can improve the level of youth off-campus sports [26]. The prior study [27] believes that the release of parental anxiety is associated with parent–child sport. Therefore, parental awareness of DBR can promote youth off-campus sports through the alleviation of parent–child relationship anxiety.

Under the increasing pressure and burden of schooling, teenagers may become resistant to learning. And when this situation happens, parents will become anxious about their children’s learning attitudes. DBR is conducive to building the internal motivation of young people and correcting their own learning attitudes, as well as helping parents to change their parenting style and adjust their expectations, thereby easing their anxiety about their children’s attitudes to learning. CCTV’s survey also showed that after the implementation of DBR, most parents’ educational anxiety had been alleviated, reducing their anxiety about their children’s learning attitude [13]. The alleviation of parental anxiety about their children’s learning attitudes may facilitate the development of a scientific educational philosophy, create a favorable family sporting atmosphere, and promote active participation of youth in off-campus sports.

Our results indicated that parental anxiety about their children’s future development and school choice did not have the mediating effects described above. This may be due to entry into high school being selected and classified according to the results of the secondary school entrance examination at the present stage in China [28]. So DBR may not relieve anxiety about school selection and future development.

### Moderating effects of parental attitudes toward their children’s exercise

Parental attitudes toward their children’s participation in sports under DBR did not significantly affect youth off-campus sports, perhaps due to the fact that parents received information about DBR that required additional cognitive processing. It would effectively promote off-campus sports for adolescents when parental education anxiety is reduced. The moderating effects obtained in this study further reinforce the view that parental attitudes toward their children’s participation in physical activity can positively moderate the effect of education anxiety on youth off-campus sports. In other words, youth off-campus sports are more likely to occur under the dual effect of reduced parental education anxiety and positive parental attitudes toward their children’s exercise. Moreover, the role of parental education anxiety alleviation in promoting youth off-campus sports was stronger under the condition of high levels of parental attitudes and weaker under the condition of low levels of attitudes. Some studies [29] have also found that the more positive the parents’ attitude towards sports, the better the attitude towards their children’s participation in sports.

## Conclusion

Parental awareness of DBR has a positive impact on youth off-campus sports. Parents’ anxiety about their children’s academic achievement, parent–child interaction anxiety, and learning attitude anxiety mediate in the process model. And relieving these anxieties will be the key to improving youth off-campus sports under the background of DBR. Parents’ attitudes toward students’ exercise can magnify the relationship between parental education anxiety and children’s off-campus sports. These have reference significance for further promoting youth sports in the context of DBR.

## Data Availability

The datasets used and/or analyzed during the current study are available from the corresponding author on reasonable request.

## Abbreviations

DBR: “Double Reduction” policy
